# Trivalent Cations Detection of Magnetic-Sensitive Microcapsules by Controlled-Release Fluorescence Off-On Sensor

**DOI:** 10.3390/nano11071801

**Published:** 2021-07-10

**Authors:** Bo-Wei Du, Ching-Chang Lin, Fu-Hsiang Ko

**Affiliations:** 1 Department of Materials Science and Engineering, National Yang Ming Chiao Tung University, Hsinchu 30010, Taiwan; duu.mse04g@nctu.edu.tw; 2 Research Center for Advanced Science and Technology (RCAST), The University of Tokyo, Tokyo 153-8904, Japan; Lin@dsc.rcast.u-tokyo.ac.jp

**Keywords:** W/O/W double emulsion, controlled-release fluorescence probe, magnetic iron oxide nanoparticles, magnetic-sensitive microcapsule, trivalent metal ion detection

## Abstract

A pyrene-based derivative, 2-((pyrene-1-ylmethylene)amino)ethanol (PE) nanoparticle, was encapsulated via water-in-oil-in-water (W/O/W) double emulsion with the solvent evaporation method by one-pot reaction and utilized as a fluorescence turn-on sensor for detecting Fe^3+^, Cr^3+^, and Al^3+^ ions. Magnetic nanoparticles (MNPs) embedded in polycaprolactone (PCL) were used as the magnetic-sensitive polyelectrolyte microcapsule-triggered elements in the construction of the polymer matrix. The microcapsules were characterized by ultraviolet–visible (UV–Vis) and photoluminescence (PL) titrations, quantum yield (Φ_f_) calculations, ^1^H nuclear magnetic resonance (NMR), scanning electron microscopy (SEM), and superconducting quantum interference device magnetometry (SQUID) studies. This novel responsive release of the microcapsule fluorescence of the turn-on sensor for detecting trivalent cations was due to the compound PE and the MNPs being incorporated well within the whole system, and an effective thermal and kinetic energy transfer between the core and shell structure efficiently occurred in the externally oscillating magnetic field. The magnetic-sensitive fluorescence turn-on microcapsules show potential for effective metal ion sensing in environmental monitoring and even biomedical applications. Under the optimal controlled-release probe fluorescence conditions with high-frequency magnetic field treatment, the limit of detection (LOD) reached 1.574–2.860 μM and recoveries ranged from 94.7–99.4% for those metals in tap water.

## 1. Introduction

Aggregation-induced emission enhancement (AIEE) probes have received significant attention in various chemical, biological and environmental applications [1,2,3,4,5]. These fluorescent probes are mainly crystalline, and their crystal structures are easily damaged by external forces, readily autoxidized, and exhibit increased background fluorescence upon sustained exposure to visible light [6,7]. Microcapsules, as effective drug-delivery system, have been widely used to incorporate various contents, such as bioactive ingredients [8,9], nanomaterials [10,11], color compounds [12,13], charged ions [14], and nanoparticles [15,16,17]. By modifying the microencapsulation method and the polymeric/monomeric material used [18], appropriate microencapsulated products for specific purposes can be achieved. Microencapsulation technology is widely used in the pharmaceutical industry [19], cosmetics [20], the agricultural industry [21], food technology [22], and the textiles industry [23]. The Schiff-based microcapsule reported by Zhipeng et al. [24], was used to mimic the cellular protrusion of filopodia. The encapsulated systems can also be applied to encapsulate fluorescent probe molecules [25]. Postiglione et al. used this system to prevent unwanted fluorescence emission from intact microcapsules when encapsulated fluorescent liquid was released from the core of ruptured microcapsules [26]. However, there are few studies on encapsulated selective fluorescent sensors or metal ion sensing; and for more general applicability, the methods of microencapsulation and other types of matrices still need to be discussed, which is the objective of this paper.

Many mechanisms can be applied to trigger the release of encapsulated core materials by chemical or physical methods, including pH [27], certain chemicals [28], light [29], ultrasonication [30], stress [31], temperature [32], and magnetic fields [33]. Among these methods, magnetic fields provide attractive characteristics, since they can effectively activate MNPs in a controllable manner through non-contact stimulation [34,35], and achieve high sensitivity and rapid-response release [36] compared with other methods. Superparamagnetic nanoparticles can effectively avoid aggregation and are evenly distributed because there is no magnetic hysteresis between them [37]; meanwhile, the thermomagnetic effect provides heating compared with the non-magnetic materials [38]. In previous studies, a high-frequency magnetic field (HFMF) was developed as a trigger to release the functional core materials from magnetic-based microcapsules [39,40,41,42]. To date, only a few attempts have been reported on the release mechanism when using heat to realize drug release by employing HFMF, which affects the thermodynamics of the microcapsule molecular chains.

Among various metal ions, Fe^3+^/Cr^3+^/Al^3+^ ions are known to be of crucial importance because of their significant pollution and their undesirable biological and environmental effects. Al^3+^ is a key factor involved in the impairment of neurotransmission leading to brain toxicity [43,44], and its concentration has a substantial impact on breast cancer [35], and metabolic disorders [45]. On the other hand, Fe^3+^ may cause damage to nucleic acids and proteins in living cells by enhancing the output of reactive oxygen species (ROS) [46]. Moreover, the industrial wastewater and gas are the main sources of chromium pollution; interestingly, Fe^3+^ has been reported to act in rocks and soils as a natural oxidizing agent for the redox transformation of Cr^3+^ into Cr^6+^ [47]. Cr^3+^ has been considered one of the top 16th toxic pollutants because of its carcinogenic and teratogenic effects on humans [48]. Compared with most of the sensing methods, fluorescent “off-on” sensors have the advantages of quick response time, low cost, high selectivity, and sensitivity for trivalent metal cations and are widely used in many applications.

**Figure 1:**
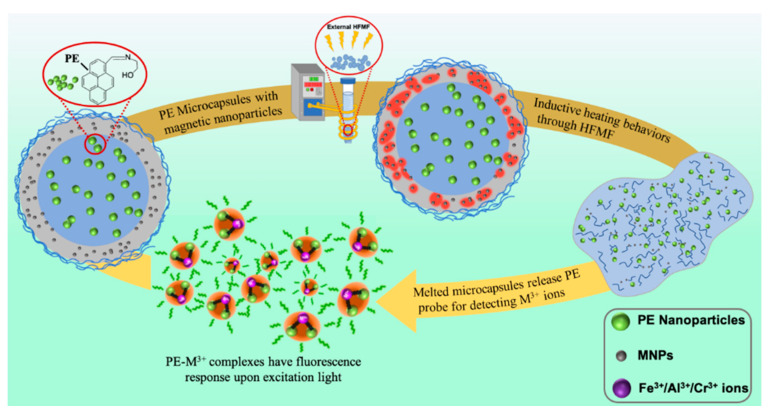
Mechanism of action of magnetic-sensitive microcapsules for sensing trivalent cations.

Although there are some pyrene-based derivative probes for the detection of various metal ions, as far as we know, the fluorescent probe PE (2-((pyrene-1-ylmethylene)amino)ethanol) and other pyrene-based derivative probes are unstable cations sensors, and they should be kept without a humid environment and visible light. Therefore, encapsulation is a possible pathway to overcome such problems. Herein, we present a novel magnetic-inductive microcapsule is designed through the use of HFMF to trigger the release of a pyrene Schiff base fluorescence probe PE from the microcapsule. The newly synthesized magnetic-sensitive microcapsules dispersed in CH_3_CN/H_2_O (9/1 *v*/*v*) underwent hysteresis heating through direct exposure to an oscillating magnetic field, which resulted in the controlled rupture of the shell material. In our previous work [49], this simple and effective fluorescent probe for the detection of trivalent cations was synthesized and fully investigated. The “off-on” fluorescent sensor selectivity towards M^3+^ (M = Fe/Cr/Al) ions was embedded into the microcapsules to achieve a controllable release behavior in response to HFMF (Figure 1) due to its photoinduced electron transfer (PET) and excimer formation in the presence of M^3+^ ions.

## 2. Materials and Methods

### 2.1. Materials

2-Aminoethanol, polycaprolactone (PCL, M_w_ = 80,000), hydrogen chloride, methanol, and ethanol were obtained from Sigma-Aldrich. Poly (vinyl alcohol) (PVA, M_w_ = 31,000–50,000), pyrene-1-carboxaldehyde, iron chloride hexahydrate, and iron chloride tetrahydrate were obtained from Alfa Aesar. Dichloromethane (DCM), acetonitrile and sodium hydroxide were purchased from J.T Baker, ECHO, and Showa, respectively. PCL was used as the microcapsule matrix material because of its good biodegradability and biocompatibility and because it can be decomposed into CO_2_ and H_2_O and then metabolized or absorbed by the organisms. The tap water was collected in our laboratory and was tested without further pretreatment.

### 2.2. Preparation of Magnetic Nanoparticles (MNPs)—Fe_3_O_4_

Fe_3_O_4_, as the magnetic nanoparticles, mentioned of MNPs throughout this article, were synthesized in deionized water. In short, a mixture of FeCl_2_·4H_2_O (0.8 g) and FeCl_3_·6H_2_O (1.04 g) in a 1:2 molar ratio was dissolved in 10 mL deionized water. Then, aqueous HCl (10.34 mL of 3.29% HCl) was added and stirred for 30 min. The solution was added dropwise to 3.0 M NaOH (50 mL) under vigorous stirring, and the black precipitates were isolated by a strong magnet and washed three times with deionized water. The MNPs were separated by centrifugation and then dried in a vacuum oven for 10 h at 30 °C.

### 2.3. Preparation of Compound PE (2-((Pyrene-1-Ylmethylene)Amino)Ethanol)

The fluorescence probe PE was fabricated through one-pot reaction. 1-equiv. of 2-aminoethanol dissolved in 50 mL of methanol was mixed with 1-equiv. of pyrene-1- carboxaldehyde under constant stirring under nitrogen and then refluxed for 12 h at 70 °C. Then, the solvent was removed through evaporation to result in the final product as a dark yellow solid.

### 2.4. Preparation of Magnetic-Sensitive PE Microcapsules

The most representative method of encapsulation is shown below, 200 mg of PE was added to 15 mL of 1.0 wt% aqueous PVA solution, resulting in the first water phase [50]. This solution was added to a mixture containing 1.50 g of PCL and 100 mg MNPs in 30 mL of DCM. Then, the mixture was stirred at 2500 rpm for 15 min, resulting in the first W/O emulsion. The mixture was added quickly into 150 mL of 1.0 wt% PVA solution under 200 rpm stirring at 40 °C for 10 min to form the W/O/W emulsion system. The products were separated by centrifugation at 2000 rpm for 5 min. Then, the products were washed with deionized water three times, and a strong magnet was used to remove the excess MNPs. The final product was dried in a vacuum oven for 10 h at 30 °C and obtained as a faint yellow powder.

### 2.5. Characterization of PE/MNPs Microcapsules

The morphology of Fe_3_O_4_ nanoparticles and PE/MNPs microcapsules were determined via scanning electron microscope (SEM, HITACHI, SU8010, Tokyo, Japan). The fluorescent samples were viewed by an OLYMPUS BX51 (Tokyo, Japan) fluorescent microscope using WU filter (excitation wavelength at 330–385 nm, emission wavelength at 430 nm) with OLYMPUS Mercury Lamp House 100 W light source under 40× magnification. Energy-dispersive X-ray spectroscopy (EDS, HORIBA, EMAX-ENERGY, Kyoto, Japan) was used to analyze the Fe_3_O_4_ nanoparticles distribution under SEM. X-ray diffraction signals of nanoparticles and functionalized microcapsules were achieved by X-ray diffraction (XRD, Bruker, D2 PHASER, Billerica, MA, USA). Dynamic light scattering (DLS, BECKMAN COULTER, Delsa Nano C, Brea, CA, USA) was used for estimating the size distribution and zeta potential of Fe_3_O_4_ nanoparticles. The size distribution was calculated by ImageJ (National Institutes of Health (NIH), Bethesda, MD, USA). The superconducting quantum interface device vibrating sample magnetometer (SQUID, MPMS-3, Quantum Design, San Diego, CA, USA) was used to obtaining the magnetic properties of Fe_3_O_4_ nanoparticles and PE/MNPs microcapsules.

### 2.6. PE Molecule Loading and Release

In this investigation, PE molecule release behavior was externally stimulated by an HFMF of 16.5 kW. The HFMF system included a cooling system, an HFMF generator, and eight loops of copper coil, and the strength of the magnetic field (H) was 2.5 kA/m. PE was loaded into the microcapsules by the double emulsion method with solvent evaporation and diffused into the solution of interest to detect the trivalent cations. To measure the concentration of the released PE molecule, 10 mL of CH_3_CN containing 2 mg PE/MNPs microcapsules was placed in a centrifuge tube in the center of the coil to apply the HFMF, where the temperature was measured by an ethanol thermometer.

## 3. Results and Discussion

### 3.1. Synthesis and Characterization of PE/MNPs Microcapsules

**Figure 2:**
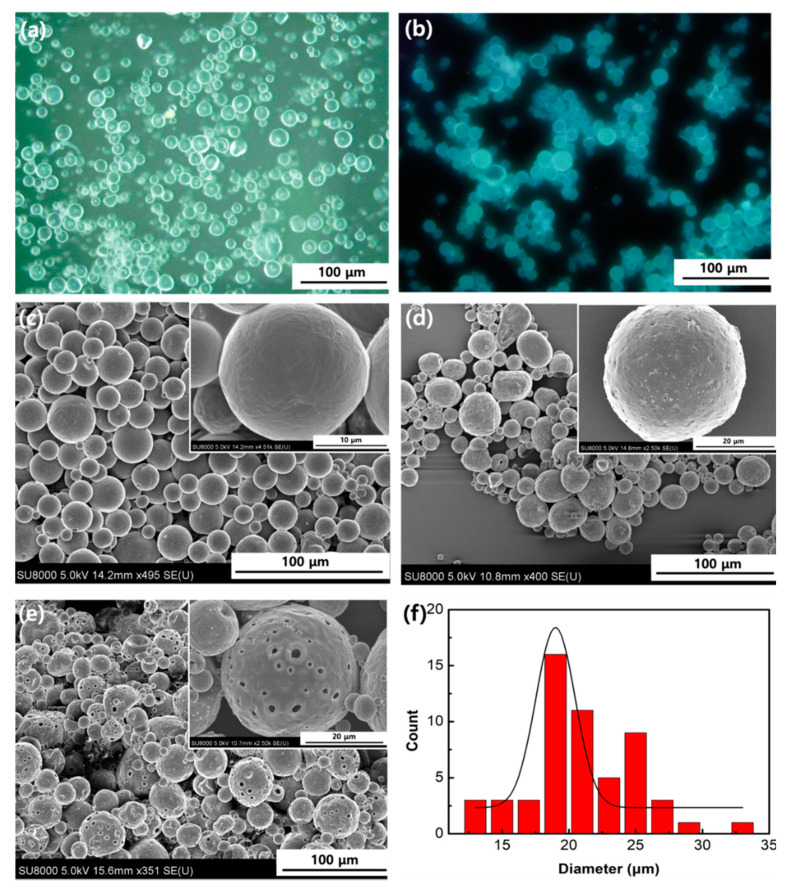
(**a**) Optical microscope image of 2-((pyrene-1-ylmethylene)amino)ethanol/magnetic nanoparticles (PE/MNPs) microcapsules; fluorescence image of (**b**) a cluster of PE/MNPs microcapsules; scanning electron microscopy (SEM) images of (**c**) hollow microcapsules and a single hollow microcapsule (inset); (**d**) a cluster of PE/MNPs microcapsules and a single PE/MNPs microcapsule (inset); (**e**) porous microcapsules under high centrifuging speed. (**f**) particle size distribution histogram of PE/MNPs microcapsules in (**c**).

**Figure 3:**
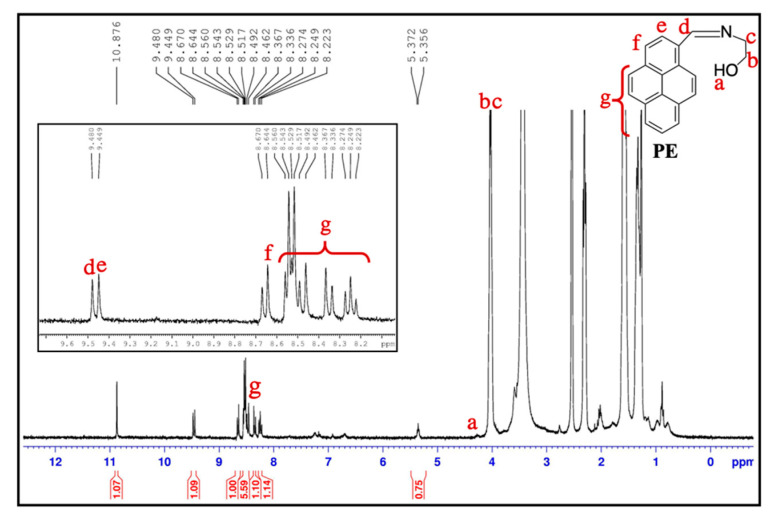
^1^H nuclear magnetic resonance (NMR) spectra of PE/MNPs microcapsules in DMSO.

In this study, microencapsulation was adopted by the solvent evaporation method using W/O/W double emulsion. The compound PE was dissolved in CH_3_CN, resulting in the first water phase, and the PCL was dissolved in DCM, resulting in the oil phase. DCM was evaporated during this procedure due to its high volatility and low boiling point. The optical microscope image of the PE/MNPs microcapsules (Figure 2a) shows that those microcapsules were distributed homogeneously and had a uniform shape with an average size of 25 ± 5 µm. A strong blue-colored emulsion of the PE/MNPs microcapsules was observed under a fluorescence microscope, which also proved that the PE/MNPs microcapsules had properties similar to property as compound PE (Figure 2b). Interestingly, the surface of the PE/MNPs microcapsules was rougher than the surface of the hollow microcapsules (Figure 2c,d), which could be related to the presence of the MNPs with a swollen structure and provide the rough surface. The microcapsules size distribution which was calculated from Figure 2c by ImageJ, the mean diameter of the microcapsules was 20.96 μm (Figure 2f). The core material was further evaluated by ^1^H nuclear magnetic resonance (NMR) titrations for confirmation of the binding sites confirmation (Figure 3). The ^1^H NMR spectra of both PE (Figure S1) and PE/MNPs microcapsules showed similar binding site positions because of PE. For clearly understanding of the peak positions of the PE compound, a–g were represented for the peaks in chemical structure, respectively. The inset on the top of this figure shows the ^1^H peaks of PE from d–g. As can be seen in this figure, the a and b peaks are not similar to the a, b peaks in the pure PE compound, due to the influence of the C–H bond. Furthermore, the centrifugation speed is one of the main factors when forming fine microcapsules. Increasing the centrifuging speed from 2000 to 4000 rpm/min resulted in a change from smooth and uniform microcapsules to porous microcapsules. The porous structure of the microcapsule shells occurred because after the microcapsules were solidified, the higher centrifugation speed caused a dispersion of the MNPs to the surface layer of the microcapsules, and some heavier nanoparticles were pushed through the shell structure (Figure 2e). Using this method to obtain the porous microcapsules is different from the traditional methods, and few studies have been reported on this topic.

**Figure 4:**
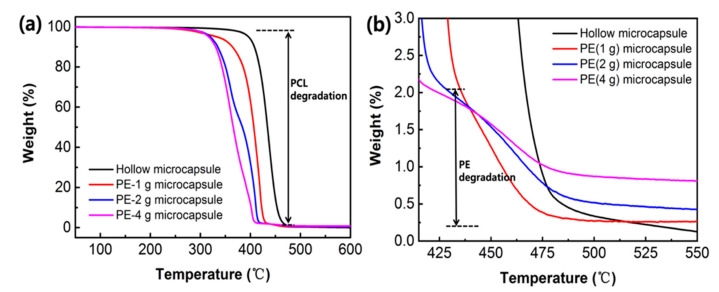
TGA curve of (**a**) the microcapsules with different amounts of core material and (**b**) the heating temperature from 412.5 to 550 °C of these microcapsules at 10 °C/min under an N_2_ flow.

In addition, the amount of PE in the microcapsules was also assessed using TGA analysis at a heating rate of 10 °C/min (Figure 4a,b). The weight reduction of these samples between 308 to 480 °C was caused by the degradation of the core and shell materials (Figure 4a), and masses of 1 g, 2 g and 4 g PE sol (5 × 10^−3^ M in CH_3_CN) as the water phase were encapsulated in the cores of the microcapsules (the hollow microcapsules acted as the control group). Figure 4b shows that some residual remained in the core at 550 °C because of the different masses of PE initially encapsulated in the core of the microcapsules, and the residual mass corresponded to the quantity of the encapsulated PE. Capsules containing different amounts of PE showed similar thermal degradation behavior from 412 to 475 °C, and the 4 g (PE solution)-loaded microcapsules showed the largest residual mass contrast compared with the 1 g (PE solution)-loaded microcapsule.

### 3.2. Magnetic Properties of the Microcapsules

**Figure 5:**
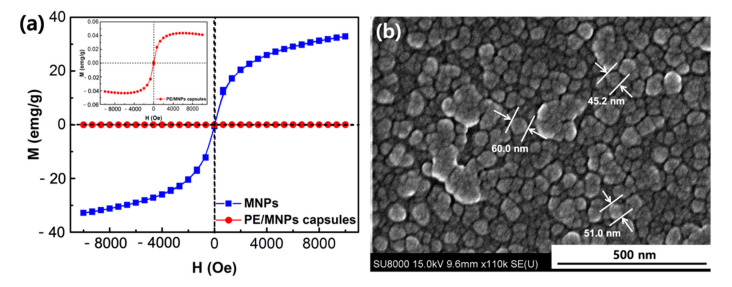
(**a**) Hysteresis curve of MNPs and PE/MNPs microcapsules. (**b**) SEM image of MNPs.

The magnetic properties of the PE/MNPs microcapsules were investigated by a SQUID at 25 °C with the magnetic field sweeping from −10,000 to +10,000 G. To verify the MNPs-loaded microcapsules, we used less amount of MNPs (30 mg) encapsulated in PE/MNPs microcapsules, the magnetizations of the MNPs and the PE/MNPs microcapsules after application of the magnetic field, as shown in Figure 5a. The inset on the top left corner showed similar tendencies; however, the PE/MNPs microcapsules showed a lower saturation magnetization (Ms), 0.043 emu/g, than the MNPs, 32.8 emu/g. This decreased magnetic strength of the microcapsules can be attributed to the amount of MNPs being much larger than the amount of MNPs encapsulated in the PE/MNPs microcapsules. Although the MNPs were well-dispersed in the oil phase during the second emulsion process, some large aggregated nanoparticles still precipitated to the bottom of the vessel because of their attractive interaction. In addition, the hysteresis curve shows the superparamagnetic properties of the MNPs and the PE/MNP microcapsules.

The SEM images confirmed that the MNPs exhibited the morphology of nanoparticles with an average particle size of 85.7 nm was measured through DLS and that they were well-dispersed instead of being aggregated (Figure 5b and Figure S2a). Its dispersion properties had also been studied using DLS such as zeta potential (ζ), mobility in very low concentration (1 µM in H_2_O; pH 7.0) (Figure S2b). The average ζ of MNPs (−33.52 mV), provides an illustration of the MNPs possessing high electron-negative value to remain stable in solvent (|ζ| > 30 mV) [51]. The X-ray diffraction pattern of MNPs was measured in Figure S3, and the EDS and elemental quantitative data were confirmed the presence of iron nanoparticles (Figure S4). In addition, the selected area diffraction pattern of the PE/MNPs microcapsules showed five planes, which are [220], [311], [400], [511], and [440], confirming the existence of iron oxide nanoparticles [52]. This also suggests that the iron oxide nanoparticles were successfully encapsulated by microcapsules (Figure S5).

### 3.3. PE Release Behaviors with High-Frequency Magnetic Field (HFMF) Treatment

**Figure 6:**
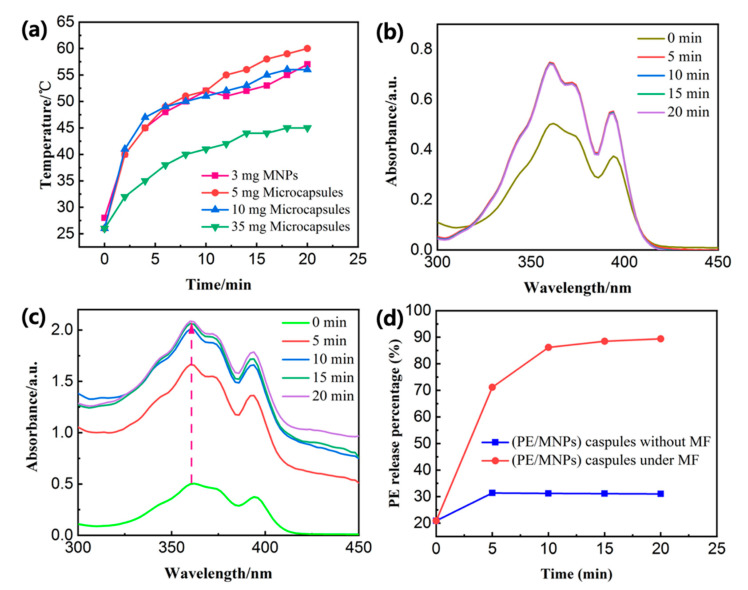
(**a**) Inductive heating ability of the different weights of the PE-loaded magnetic nanoparticles microcapsules (PE/MNPs microcapsules) under high-frequency magnetic field (HFMF) exposure (16.5 kW). (**b**) PE molecule release of PE/MNPs microcapsules (5 mg) in 1.5 mL CH_3_CN without HFMF at room temperature monitored by ultraviolet–visible (UV–Vis) spectroscopy. (**c**) PE molecule release profile of PE/MNPs microcapsules in 1.5 mL CH_3_CN under 20 min HFMF exposure (16.5 kW). (**d**) PE molecule release behaviors of PE/MNPs microcapsules under HFMF and room temperature for 20 min.

While the PE/MNPs microcapsules under HFMF exposure, a significant increase in the temperature of inductive heating is illustrated in Figure 6a. The results show that 5 mg and 10 mg microcapsules had a similar rising trend to 3 mg pure MNPs, the bulk solution temperature increased to 58 ± 2 °C and 57 °C from the room temperature due to the hyperthermia effect within 20 min. Meanwhile, the 35 mg microcapsules exhibited lower temperature (45 °C) after the same time stimulus because more liquid needed more power to apply. The cumulative PE molecule release of PE-loaded magnetic microcapsules at room temperature and under HFMF exposure was investigated by ultraviolet–visible (UV–Vis) spectroscopy (Figure 6b,c). The cumulative arrived to 2.056 a.u. after 20 min of HFMF exposure; on the other hand, cumulative release of PE from PE/MNPs microcapsules at room temperature without any treatment from outside was only 0.714 a.u. The amount of PE was release from the microcapsules with HFMF treatment was almost three times higher than the control group. The PE molecule release behavior was also observed for the characteristic peaks of PE in UV–Vis spectrum such as 358 nm. In the absence of the magnetic field (MF), the PE only released 31.39% at most, however, the PE molecule release percentage increased sharply from 20.91% to 89.39% after 20 min stimulus (Figure 6d). It seems that in the first 5 min of stimulus, the PE had the biggest release rate (from 20.91% to 71.13%), then the release rate growth slows down, proved that the shell structure of microcapsules dissociated to a great extent. According to Figure 6a, the PE could release 71.13% under the environmental temperature around 45 °C, however, it is believed that the temperature around the MNPs is much higher than the surrounding temperature. This result strongly illustrates that the content of the core material (PE) released from the PE/MNPs microcapsules after HFMF stimulus as large as about three times compared to that without the magnetic field applied.

**Figure 7:**
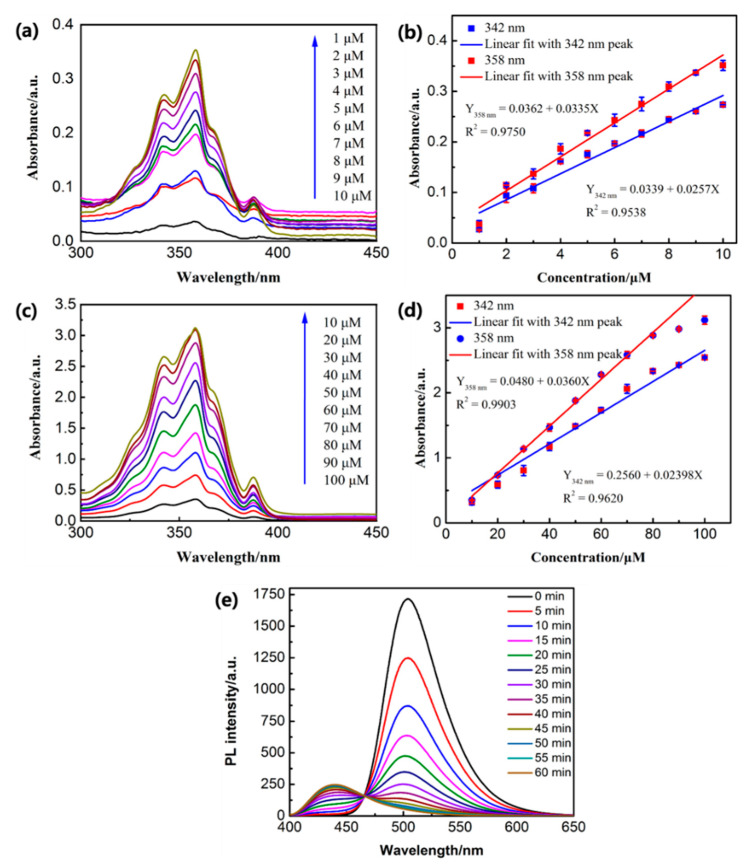
(**a**,**c**) UV–Vis absorption spectral changes of PE with different ranges of concentration (from 1–10 µM) and (from 10–100 µM). (**b**,**d**) linear regression corresponding to the different range of concentration showing the absorbance peaks at 342 nm and 358 nm. (**e**) PL titrations of PE (100 µM) in CH_3_CN with Fe^3+^ were exposed in air within 60 min.

To further elucidate the release behavior of the PE/MNPs microcapsules under the HFMF stimulus, a quantitative estimation of the amount of PE in CH_3_CN was obtained by a UV-Vis standard calibration curve. To measure the concentration of PE molecule release, 1.5 mL of CH_3_CN with 4 mg dispersed PE/MNPs microcapsules was stimulated by an oscillating magnetic field at 25 °C. In the UV–Vis spectra, in response to the magnetic field, a redshift was observed in the peak of the non-encapsulated pure PE, and the peaks of the PE/MNPs microcapsules at 342/358 nm also redshifted to 361/372 nm. The two absorbance peaks at 342 nm and 358 nm in the different concentration ranges (1–10 µM) and (10–100 µM) (Figure 7a–c) could be linearly fitted (R^2^ = 0.97240/0.97657 and 0.95125/0.98719) to obtain the calibration curve. These results indicate the possibility of quantitative PE detection within the good linear range (Figure 7b–d). By increasing the incubation time of PE (100 µM in CH_3_CN; pH 7.0) with Fe^3+^ ions (1 mM in CH_3_CN), it was clearly observed that the PL peak at 504 nm, in the beginning, was gradually decreased, and a new peak appeared at 439 nm, which increased in intensity after 20 min of incubation (Figure 7e). This phenomenon might be related to the fluorescence decay of PE-M^3+^ complexes; the fluorescent intensity reached its maximum value in the beginning and decreased with increasing time.

### 3.4. Sensor Titrations of M^3+^ (M = Fe/Cr/Al) under HFMF Stimulus

**Figure 8:**
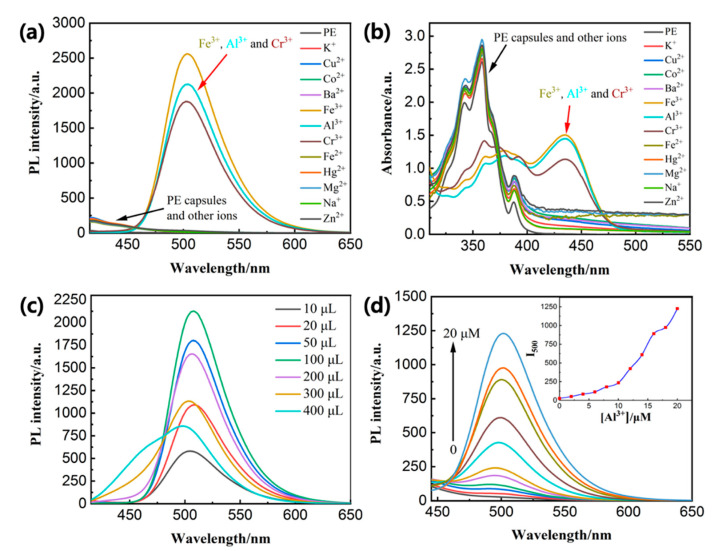
(**a**,**b**) Fluorescence responses (λ_ex_ = 395 nm) and UV–Vis responses of the released PE/MNP microcapsules (2 mg in 10 mL CH_3_CN) towards 50 µM of various metal ions in H_2_O. (**c**) PL spectra of released PE/MNPs microcapsules (2 mg in 10 mL CH_3_CN) as a function of the increasing volume of 50 µM Al^3+^ in water (λ_ex_= 395 nm) from 10 to 400 µL. (**d**) PL (λ_ex_ = 395 nm) titrations of released PE/MNP microcapsules (2 mg in 10 mL CH_3_CN) with Al^3+^ in water with an equal span of 2 µm; PL inset: intensity changes at 500 nm towards Al^3+^ concentration.

**Figure 9:**
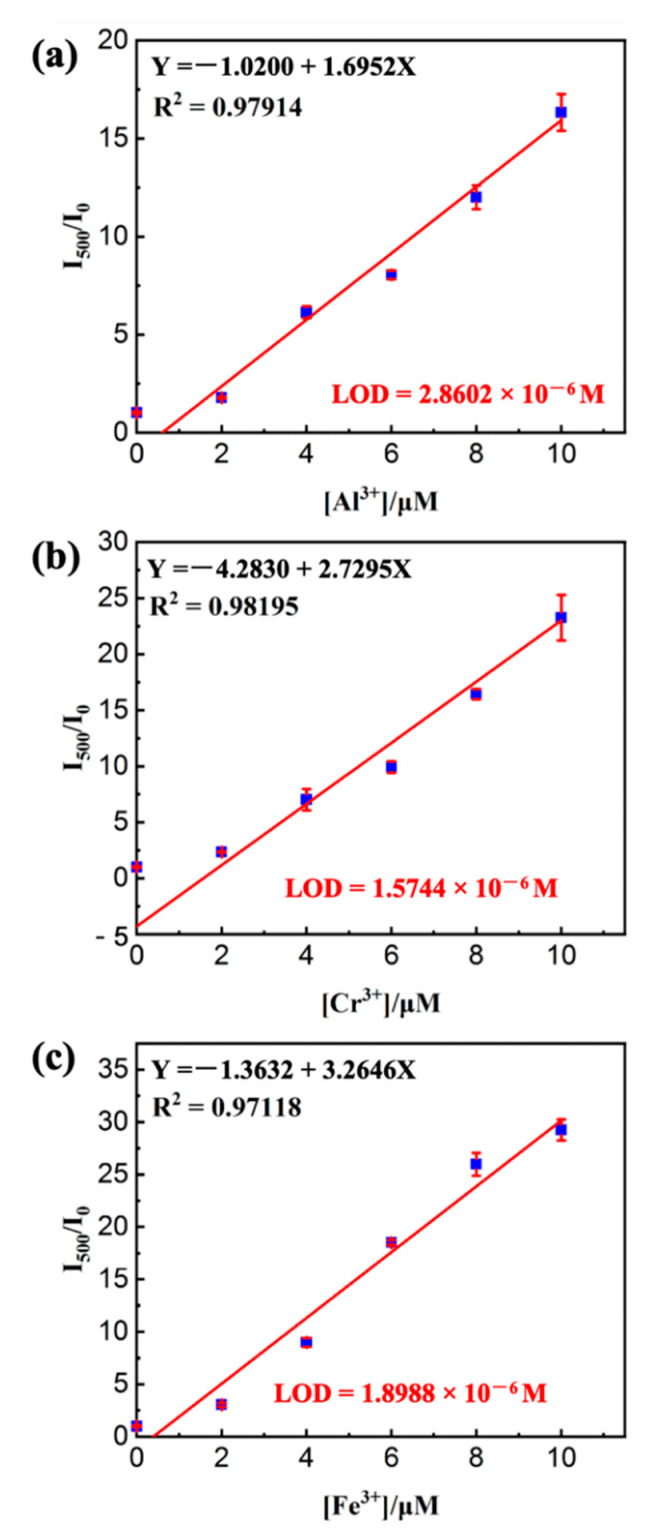
Standard deviation and linear fitting calculations for detection limits of (**a**) PE/MNPs microcapsules—Al^3+^ (**b**) PE/MNPs microcapsules—Cr^3+^ and (**c**) PE/MNPs microcapsules—Fe^3+^ based on PL intensity changes at 500 nm.

Because of the AIEE of the PE probe, the sensing ability of PE/MNPs microcapsules was evaluated in CH_3_CN/H_2_O (9:1 *v*/*v*), and to avoid confusion between the AIEE and sensor selectivity, all metal ions (K^+^, Ba^2+^, Cu^2+^, Co^2+^, Al^3+^, Cr^3+^, Fe^3+^) concentrations were 50 µM in deionized water, as described below. Therefore, for the sensor titrations, 2 mg of PE/MNPs microcapsules in CH_3_CN (10 mL) was investigated with 50 µM of metal ions in deionized water. In addition, the concentration of PE in 2 mg PE (10 mM)/MNPs microcapsules is equivalent to the 50 µM pure PE compound was noticed, thus all results were established based on the weight of PE/MNPs microcapsules taken as 2 mg, as explained above. Upon the application of a magnetic field for 20 min, the PE molecule released from the microcapsules was demonstrated in Figure 8a,b, the released PE/MNPs microcapsules displayed impressive selectivity to Al^3+^, Cr^3+^, and Fe^3+^ (M^3+^) metal ions, to cope with these metal ions solutions and exhibited absorbance peaks (UV–Vis) and turn-on emission peaks (PL) at 435 nm and 502 nm, respectively, which showed red-shifted from their original position (PE/MNPs microcapsules; λ_abs_ = 355 nm and λ_em_ = 417 nm; Φ_f_ = 0.0094). Furthermore, according to the volume of Al^3+^ solution increased, the turn-on emission was enhanced (from 10 to 100 µL), then blue shift has taken place at its emission peak due to the influence of the solvent polarity, meanwhile, the intensity was gradually decreased (Figure 8c). By increasing the concentration of Al^3+^ (0–20 µM with an equal span of 2 µM in H_2_O) the sensitivity of PE/MNPs microcapsules (50 µM in CH_3_CN) towards Al^3+^ ions was distinctly observed in the UV–Vis spectrum as shown in Figure 8d. This fluorescence spectrum of released PE/MNPs microcapsules (λ_em_ = 417 nm) showed red shift turn-on responses at 495 nm for PE/MNPs microcapsules with Al^3+^ and inset showed the fluorescence intensity changes as a function of Al^3+^ concentration; similarly, Fe^3+^ and Cr^3+^ are also showed strong function with released PE/MNPs microcapsules as the increment of their concentration. To prove the selectivity of PE/MNPs microcapsules towards M^3+^ ions, the detection limit (LOD) [53] calculations were performed after application of HFMF for 20 min using standard deviation and linear fittings. By observing the function of M^3+^ ions concentration and the relative fluorescence intensity (I/I_0_), the detection limits of PE/MNPs microcapsules with M^3+^ complexes were estimated as 2.8602 × 10^−6^ M (PE/MNPs microcapsules-Al^3+^), 1.5744 × 10^−6^ M (PE/MNPs microcapsules-Cr^3+^) and 1.8988 × 10^−6^ M (PE/MNPs microcapsules-Fe^3+^), respectively (Figure 9).

### 3.5. Comparison of Other Probes

Although there are some pyrene-based derivative probes for the detection of various metal ions, as far as we know, the fluorescent probe PE and other pyrene-based derivative probes are unstable cation sensors. Hence, they should be kept without a humid environment and visible light. In that sense, the microcapsule system perfectly meets these conditions for better preservation. Furthermore, under the fluorescent probe delivery system, we can easily release a large dose of fluorescent sensors under certain circumstances in a very short time, the most important is our pyrene-based magnetic-sensitive microcapsules showed high selectivity and good LOD compared with other pyrene-based derivative sensors in Table 1.

To confirm the feasibility of our system in environmental monitoring and other application fields, Fe^3+^/Cr^3+^/Al^3+^ activity was investigated in three real tap water samples containing Fe^3+^, Cr^3+^ and Al^3+^ ions, respectively. Our sensor displayed excellent precision and reproducibility, as explained by the coefficients of variation (Fe^3+^ ≤ 2.79%, Cr^3+^ ≤ 2.79%, Al^3+^ ≤ 3.76%) and recoveries were in the range of 96.5–98.7% for Fe^3+^, 96.7–99.4% for Cr^3+^ and 94.7–98.9% for Al^3+^ (Table 2). These results also indicate that the system we proposed in this study is very compatible with the practical measurement of Fe^3+^/Cr^3+^/Al^3+^ ions activity in future environmental monitoring and even biomedical applications.

## 4. Conclusions

Conventional pyrene-based derivative sensors for metal ions detection face the problems of a humid environment and visible light interference. This study proposes an alternating approach that pyrene-based derivative (PE) nanoparticle was encapsulated via a double emulsion system with a solvent evaporation method in a one-pot reaction. Similar to the neat PE compound, the core material PE showed excellent AIEE nature and displayed selective turn-on fluorescence for trivalent cations (Fe^3+^/Cr^3+^/Al^3+^) detection. The MNPs effectively triggered the rupture of the shell structure through an external oscillating magnetic field, and the microcapsules were heated by the interaction of the MNPs with the magnetic field. The centrifugation speed during the synthesis process crucially affected the morphology of the microcapsules. These results were supported by UV–Vis/PL, quantum yield (Φ_f_) calculations, SEM, SQUID, and ^1^H NMR titrations. Hence, a possible sensing mechanism of the photoinduced electron transfer (PET) and excimer formation was successfully discussed. Due to the low melting point of PCL, the PE/MNP microcapsules released the core material percentage up to 89.39% under 20 min the influence of HFMF because the temperature around the microcapsules reached up to 60 °C. The results showed that PE encapsulated in microcapsules was highly stable and selective towards Fe^3+^/Cr^3+^/Al^3+^ in the presence of various metal ion solutions. The PE-M^3+^ sensor complex did not have an obvious impact on the specific performance and the LOD. The standard deviation and linear fittings for the LOD were calculated as 10^−6^ M limit for Fe^3+^, Al^3+^, and Cr^3+^ ions. This sensing platform can potentially also be used for trivalent cations’ detection in tap water.

## Figures and Tables

**Table 1:** Comparison of previous reported pyrene-based derivative sensors for metal ions detection.

Probe	Metal Ions	Source of Sample	LOD (μM)	Ref.
Aminopropyl−1-pyrenebutanamide cucurbit [10] uril	Fe^3+^	Deionized water	550	[54]
Bis(2-picolyl)amine-modified pyrene derivative with sodium dodecyl sulfate	Fe^3+^, Al^3+^, Mg^2+^, Pb^2+^, Ca^2+^ and Ba^2+^	Mineral Water Samples	50	[55]
3,3′,3′′,3′′′-(pyrene-1,3,6,8-tetrayltetrakis(sulfanediyl))tetrabenzoic acid	Pb^2+^	-	0.2	[56]
1-(3,5-dihydropyren-1-yl)-2-((1-methyl-1,6-dihydropyrimidin-2-yl)thio)ethan-1-one	Fe^3+^	Deionized water	3.06	[57]
(E)-1-(pyren-1-yl)-N-tritylmethanimine	Hg^2+^	Deionized water	0.4	[58]
1,5-dimethyl-2-phenyl-4-((pyren-1-ylmethylene)amino)-1H-pyrazol-3(2H)-one	Cu^2+^	-	2.5	[59]
1,2-bis(2-(4-(1,2,2-triphenylvinyl)phenyl)-1,3-dithiolan-2-yl)benzene	Hg^2+^	Distilled water	10	[60]
1-(pyren-1-yl)-N,N-bis-(pyridine-2-ylmethyl)methanamine	Cu^2+^, Fe^3+^	Distilled water	4.9	[61]
6-methoxy-N-(pyren-1-ylmethylene)benzo[d]thiazol-2-amine	Fe^3+^, Fe^2+^	Distilled water	2.61	[62]
2-((pyren-1-ylmethylene)amino)ethanol	Fe^3+^, Al^3+^ and Cr^3+^	Distilled water	0.106–0.117	[49]
2-((pyren-1-ylmethylene)amino)ethanol magnetic-sensitive microcapsules	Fe^3+^, Al^3+^ and Cr^3+^	Tap water	1.574–2.860	This work

**Table 2:** Determination of Fe^3+^/Cr^3+^/Al^3+^ ions in sample.

Sample	Added Fe^3+^/Cr^3+^/Al^3+^ Concentration (μM)	Detected Concentration ^a^ (μM)			Recovery ^b^ (%)		
		Fe^3+^	Cr^3+^	Al^3+^	Fe^3+^	Cr^3+^	Al^3+^
1	5	4.874 ± 0.136	4.933 ± 0.232	4.894 ± 0.184	97.4	98.7	97.9
2	20	19.739 ± 0.337	19.349 ± 0.748	18.972 ± 0.612	98.7	96.7	94.7
3	50	48.241 ± 0.619	49.721 ± 1.281	49.434 ± 0.941	96.5	99.4	98.9

^a^ Mean value and standard deviation of three measurements. ^b^ Detected value/spike conc.

## Data Availability

Data sharing not applicable.

## Supplementary Materials

The following are available online at https://www.mdpi.com/article/10.3390/nano11071801/s1. Figure S1.^1^H NMR of PE, Figure S2. Zeta potential measure of magnetic nanoparticles (MNPs), Figure S3. The X-ray diffraction pattern of MNPs. Figure S4. The energy-dispersive X-ray (EDS) spectrum and elemental quantitative data of PE/MNPs microcapsules. Figure S5. Characterization analysis of PE/MNPs microcapsules and hollow microcapsules.
