# Supplementary material for: Trivalent Cations Detection of Magnetic-Sensitive Microcapsules by Controlled-Release Fluorescence Off-On Sensor
Source: Nanomaterials (Basel). 2021 Jul 10;11(7):1801. doi: 10.3390/nano11071801 (PMC8308368; doi:10.3390/nano11071801)
Supplement: Supplementary file 1 [file nanomaterials-11-01801-s001.zip › nanomaterials-1287172-supplementary.pdf]

nanomaterials

MDPI

Supplementary Materials

Trivalent Cations Detection of Magnetic-Sensitive Microcapsules by Controlled-Release Fluorescence Off-On Sensor

Bo-Wei Du [1], Ching-Chang Lin [2] and Fu-Hsiang Ko [1,*]

1 Department of Materials Science and Engineering, National Yang Ming Chiao Tung University, Hsinchu 30010, Taiwan; duu.mse04g@nctu.edu.tw
2 Research Center for Advanced Science and Technology (RCAST), The University of Tokyo, Tokyo 153-8904, Japan; Lin@dsc.rcast.u-tokyo.ac.jp
* Correspondence: fhko@mail.nctu.edu.tw; Tel.: +886-35712121 (ext. 55803)

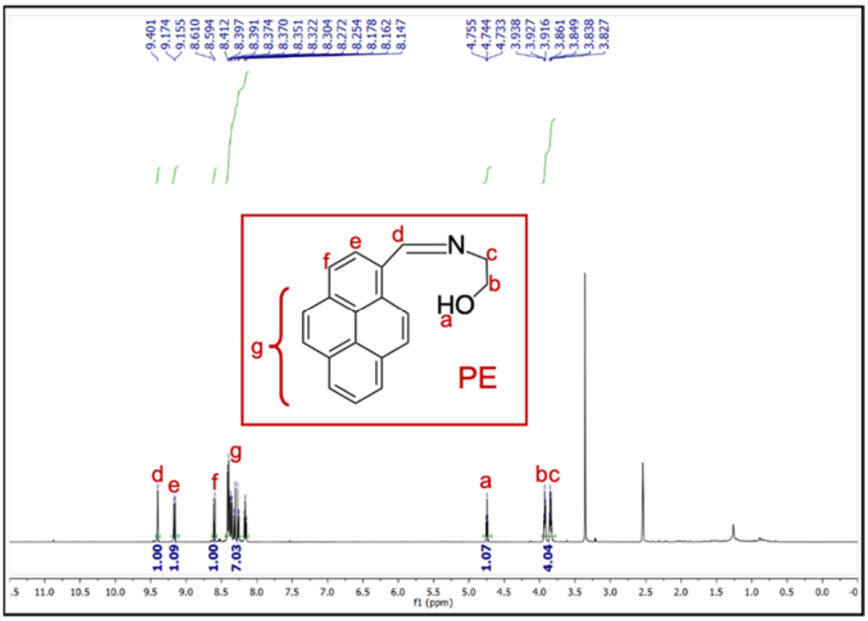

Figure S1. ^{1}H NMR spectrum of PE in DMSO.

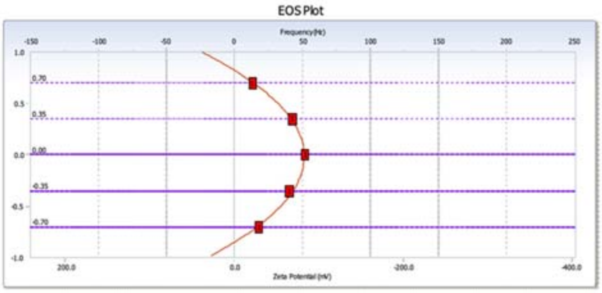

Measurement Results

Zeta Potential	: -33.52	(mV)	Doppler shift	: 20.81	(Hz)
Mobility	: -2.614e-004	(cm²/Vs)	Base Frequency	: 125.0	(Hz)
Conductivity	: 0.0211	(mS/cm)			

Figure S2. Zeta potential measure of magnetic nanoparticles (MNPs).

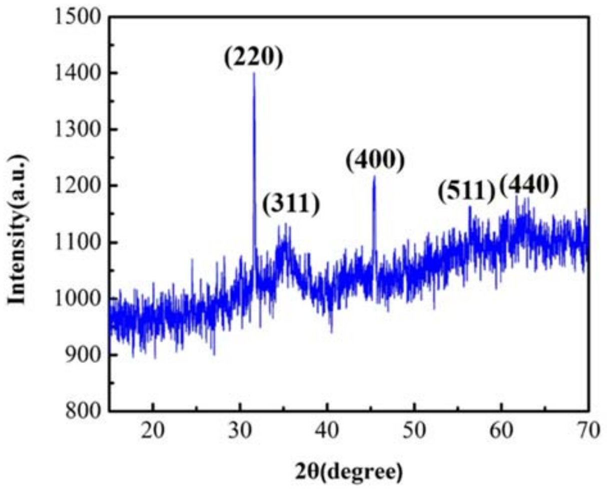

Figure S3. The X-ray diffraction pattern of MNPs.

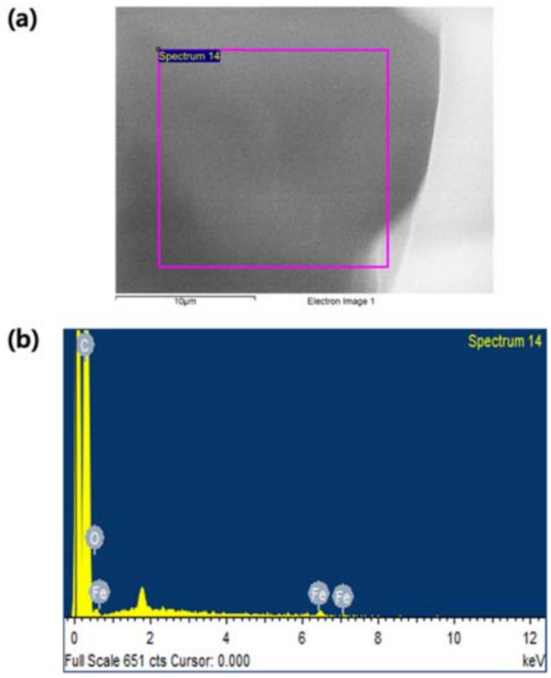

Element	Weight%	Atomic%
C k	94.07	96.89
O k	3.25	2.51
Fe k	2.68	0.59
Total	100.00	99.99

Figure S4. (a) and **(b)** The EDS spectrum and elemental quantitative data of PE/MNPs microcapsules.

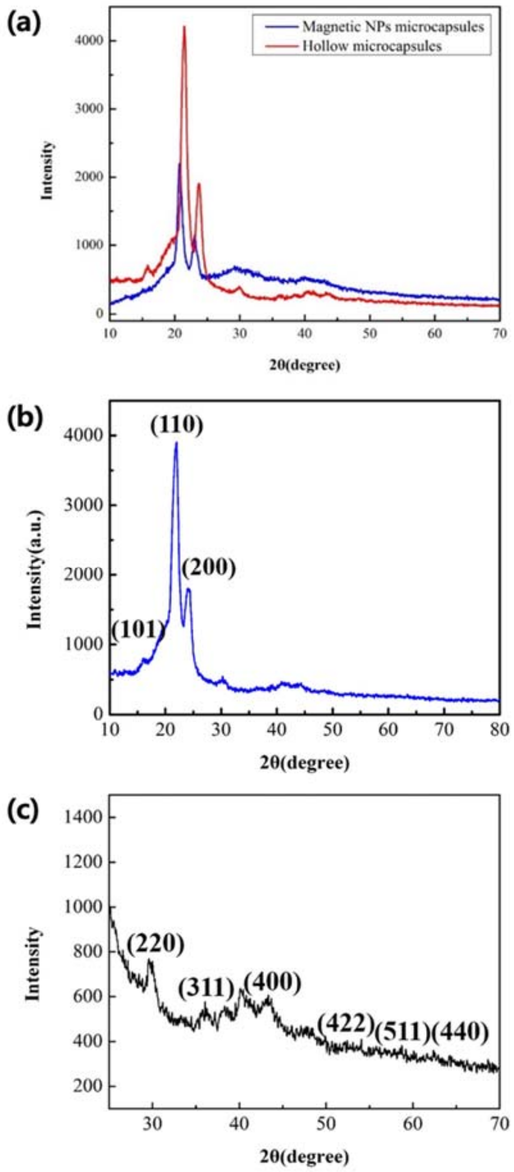

Figure S5. Characterization analysis of **(a)** PE/MNPs microcapsules and hollow microcapsules. **(b)** and **(c)** PE/MNPs microcapsules.
